# 

**Published:** 1874-06

**Authors:** 


### ACKNOWLEDGMENTS.

Atlanta Daily Herald; Rome Daily Commercial; American Journal of Pharmacy; American Practitioner; Archives of American Medicine and Surgery; Boston Medical and Surgical Journal; Boston Journal of Chemistry; Baltimore Physician and Surgeon; Baffalo Medical and Surgical Journal; Cincinnati Lancet and Observer; The Clinic; Detroit Review of Medicine and Pharmacy; Druggists' Circular; The Druggist; The Galaxy; Herald of Health; Indiana Journal of Medicine; Journal of Materia Medica; London Lancet; Medical Examiner; Medical Record; Medical News and Library; Medical and Surgical Reporter; Nashville Journal of Medicine and Surgery; New Orleans Medical and Surgical Journal; North-Western Medical and Surgical Journal; Obstetrical Journal of Great Britain and Ireland; Pacific Medical and Surgical Journal; Philadelphia Medical Times; Popular Science Monthly; Richmond and Louisville Medical Journal; St. Louis Medical and Surgical Journal; Virginia Medical Monthly.

